# Correction: Expanding medicinal chemistry into 3D space: metallofragments as 3D scaffolds for fragment-based drug discovery

**DOI:** 10.1039/d2sc90145e

**Published:** 2022-08-05

**Authors:** Christine N. Morrison, Kathleen E. Prosser, Ryjul W. Stokes, Anna Cordes, Nils Metzler-Nolte, Seth M. Cohen

**Affiliations:** Department of Chemistry and Biochemistry, University of California San Diego La Jolla CA 92093 USA scohen@ucsd.edu; Lehrstuhl für Anorganische Chemie 1, Bioanorganische Chemie, Ruhr-Universität Bochum, Universitätsstraße 150 44801 Bochum Germany

## Abstract

Correction for ‘Expanding medicinal chemistry into 3D space: metallofragments as 3D scaffolds for fragment-based drug discovery’ by Christine N. Morrison *et al.*, *Chem. Sci.*, 2020, **11**, 1216–1225, https://doi.org/10.1039/C9SC05586J.

The authors regret that in the original article, inhibitory values reported for some metallofragments were incorrect. Unfortunately, DMSO stock solutions of reportedly active ferrocene-based metallofragments were found to decompose in the presence of light, which resulted in inaccurate inhibition values. The authors maintain that the core conclusions of the paper are accurate and the utility of three-dimensional metal complexes for fragment-based drug discovery has merit.

In the original article, ‘class A’ metallofragments are comprised of ferrocene derivatives (Fig. 1). Some of these ferrocene fragments (specifically those containing carbonyl groups) are reported as broadly inhibiting several protein targets. It was noted in our original report that the ferrocene scaffold was likely promiscuous due to its lipophilicity and potential redox activity, but that it might still serve as a useful metallofragment for fragment-based drug discovery (FBDD) campaigns. However, re-evaluation of these compounds against the influenza endonuclease (PA_N_) failed to reproduce our original inhibition results for the class A metallofragments using freshly prepared stocks, indicating a problem with the materials used in the original study.

**Fig. 1:**
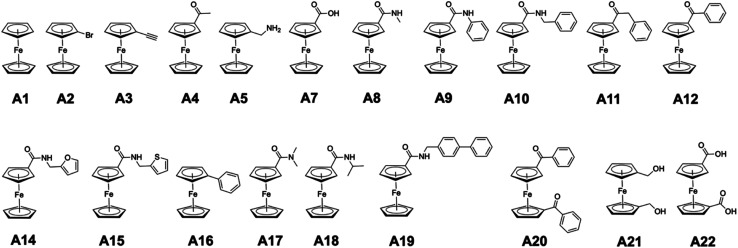
Chemical structures of class A metallofragments.

Several compounds from class A were originally reported as having near complete (100%) inhibition against PA_N_ endonuclease at an inhibitor concentration of 200 μM (Tables 1 and 2). However, when re-evaluated under identical conditions, using freshly prepared DMSO stock solutions, inhibition was only observed with one fragment of this class (A22, Fig. 1), with the previously reported highly active fragments (A4, A7–A21, Table 1) not showing significant inhibition activity. Class A metallofragments lacking carbonyl functional groups showed similarly low activity in both our original and replicated experiments. Only compound A22 retained significant inhibitory activity against PA_N_ when a fresh stock solution of the fragment was prepared, isolated from light, and immediately evaluated.

Reported (original) and corrected (this correction) percent inhibition values of class A metallofragments against PA_N_ endonuclease at an inhibitor concentration of 200 μM^a^

**Table:** 

Compound	A1	A2	A3	A4	A5	A7	A8	A9	A10	A11
Reported	12 ± 6	<1	<1	45 ± 14	8 ± 7	103 ± 5	103 ± 4	53 ± 5	46 ± 7	90 ± 5
Corrected	3 ± 10	n.d.	18 ± 3	6 ± 3	21 ± 5	9 ± 3	10 ± 5	4 ± 2	16 ± 4	10 ± 7

a n.d. = not determined.

**Table:** 


Compound	A12	A14	A15	A16	A17	A18	A19	A20	A21	A22
Reported	66 ± 5	26 ± 6	55 ± 7	19 ± 8	100 ± 4	107 ± 6	32 ± 8	80 ± 4	10 ± 16	88 ± 9
Corrected	9 ± 4	10 ± 5	18 ± 11	5 ± 6	5 ± 3	<1	11 ± 9	<1	< 1	93 ± 1

Reported and re-evaluated percent inhibition values of representative metallofragments against PA_N_ endonuclease at 200 μM inhibitor concentration. Each compound was tested in triplicate from either two or three independent experiments^a^

**Table:** 

Compound	A1	B1	C1	D1	E1	F1	G1
Reported	12 ± 6	4 ± 6	70 ± 23	20 ± 11	18 ± 9	82 ± 5	16 ± 6
Re-evaluated	<5	19 ± 8	75 ± 11	14 ± 9	<5	10 ± 14	<5

a n.d. = not determined.

**Table:** 


Compound	H1	I1	J1	K1	L1	M1	DPBA
Reported	31 ± 6	26 ± 7	25 ± 6	99 ± 3	12 ± 4	26 ± 4	n.d.
Re-evaluated	25 ± 9	<5	41 ± 6	83 ± 3	30 ± 8	54 ± 5	97 ± 1

In the original article, one representative member of each metallofragment class was assessed for stability by NMR. Compound A1 (ferrocene) proved stable in DMSO and class A metallofragments were stored as DMSO stocks at −80 °C, but were not consistently protected from light. As noted above, many of the derivatives in class A contain a ferrocenyl carbonyl motif. It has been previously reported that ferrocenyl ketones can undergo photoaquation (*λ* > 280 nm) in wet DMSO to produce a monocyclopentadienyliron cation, the anionic ligand, and free cyclopentadiene.^1^ Suspecting issues with photostability, we dissolved several of the ferrocenyl fragments in DMSO-*d*_6_, exposed them to ambient room light (fluorescent light bulb), and monitored stability by NMR. Indeed, photoinstability was confirmed by the observance of free cyclopentadienyl peaks appearing in the ^1^H NMR spectrum (Fig. 2). It should also be noted that while the fresh stock of A22 retained significant inhibition against PA_N_, it also exhibits sensitivity to light in DMSO.

**Fig. 2:**
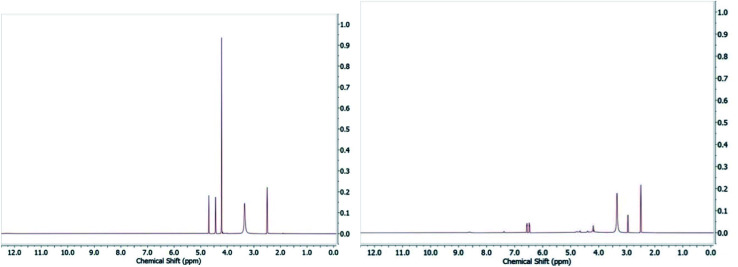
Compound A7 in DMSO-*d*_6_ (left) and after exposure to ambient light for 24 h (right) demonstrating the photoinstability of this compound.

Based on these findings, the authors regret that the inhibitory data associated with class A metallofragments are incorrect, likely because of photodecomposition of these ferrocene derivatives. To confirm if other classes of metallofragments were correctly reported, a representative member of each class was evaluated against PA_N_ endonuclease at an inhibitor concentration of 200 μM using freshly prepared DMSO stocks. Each compound was tested in triplicate in two or three independent experiments, with the addition of 2,4-dioxo-4-phenylbutanoic acid (DPBA) as a positive control.^2^ Fortunately, these experiments largely reproduced our original findings. Although several fragments showed slightly greater activity upon re-evaluation (J1, L1, M1, Fig. 3), only one fragment initially identified as a hit (>50% inhibition) failed to show activity when re-examined (F1, Fig. 3). Other than compound F1, all selected compounds designated as ‘hits’ (>50% inhibition) retained a high level of inhibitory activity upon re-evaluation. Taken together, the authors believe the inaccuracies stemming from photostability issues are limited to class A compounds; however, these inaccuracies would include all other inhibition data reported for class A compounds, including assay data against other enzyme targets, IC_50_ values, and thermal shift assay (TSA) binding data. Furthermore, the hit rate against each target is likely lower than reported, with PA_N_ having an adjusted hit rate of ∼28% (20/71).

**Fig. 3:**
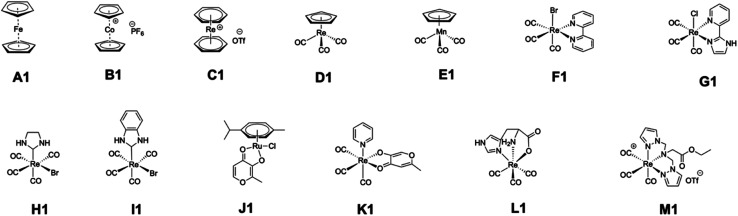
Chemical structures of representative metallofragments from each class re-examined for inhibition activity against PA_N_ endonuclease.

The authors maintain that three-dimensional metallofragments represent a useful new line of inquiry for FBDD and our ongoing studies seek to further test this hypothesis. The core message of our original study – the ability of metallofragments to be useful scaffolds for FBDD that occupy hard-to-access three-dimensional chemical space – remains unchanged. However, as demonstrated by our error, the authors acknowledge that metallofragments may pose unique challenges that must be carefully considered and controlled for when using them in FBDD campaigns.

The authors would like to take this opportunity to thank the readers who alerted them to the concerns regarding the inhibitory activities and allowed them to reinvestigate. Both the authors and the Royal Society of Chemistry appreciate their support.

The Royal Society of Chemistry apologises for these errors and any consequent inconvenience to authors and readers.

## Supplementary Material

## References

[cit1] Ali L. H., Cox A., Kemp T. J. (1973). Photochemistry of Ferrocenyl Ketones and Acids in Dimethyl Sulphoxide and Related Solvents. J. Chem. Soc., Dalton Trans..

[cit2] Tomassini J. (1994). *et al.*, Inhibition of cap (m^7^GpppXm)-dependent endonuclease of influenza virus by 4-substituted 2,4-dioxobutanoic acid compounds. Antimicrob. Agents Chemother..

